# Repertory of the Back

**Published:** 1899-07

**Authors:** E. H. Wilsey

**Affiliations:** Parkersburg, W. Va.


### REPERTORY OF THE BACK.

E. H. Wilsey, M. D., Parkersburg, W. Va.

(Continued from June number, page 295.)

### SACRUM.

**Burning** pain in abdomen when sitting bowed forward, extending into sacrum; ceasing when he straightens himself, Nitrum.

— intense in small of back and sacrum, commencing in region of kidney, passing around on both sides above hips into groin, also downward from renal region through gluteal region down back part of thigh; burning pain, ameliorated by no position, *Lac-def.*

— startling stitch in sacrum, Mur-acid.

— cutting and scraping in sacrum, Nat-c.

— in sacrum when sitting, Borax.

**Burrowing** and boring in sacrum and anus, Callad.

— sticking on left side near sacrum, Dulc.

**Carriage**, riding causes pain in sacrum, *Nux-mosch.*

**Catamenia,** pain in sacrum when sitting as if the catamenia would appear, Carbo-an.

**Chest,** pain in sacrum so violent that it drew chest together with pressure on stomach and constriction of abdomen, Lyco.

— during confinement at night a sort of spasm of chest coming from sacrum up the back, first into the gastric region, then into chest; it makes breathing difficult and causes anguish, Lyco.

— pain in sacrum during stool, with distention of abdomen, extending into chest, *Carbo-an.*

— at night pain in sacrum and stitches in both hips and in left side of chest, Lyco.

— an occasional stitch from the sacrum through left side of abdomen toward chest, Kali-c.

— at night colic for two hours with subsequent stool, then in the

SACRUM.

morning pain in sacrum and chest, and afternoon all the limbs feel bruised, Kali-c.

**Chills** run up back from sacrum to base of occiput, *Gels.*

— and cold feeling across loins and sacrum, *Lil-tig.*

— from sacrum up back, *Kali-iod.*

— attacks of stitches in sacrum that take away the breath with pain in the head and nape, followed by frequently alternating chill and heat, with anxiety about the scrobiculus cordis until evening, Sul.

**Chilliness** and pain in sacrum during menstruation, *Puls.*

— in sacrum, Lyco.

**Clucking** in right side beside the sacrum, Sepia.

— somewhat to the left upward from the sacrum, Lyco.

**Contractive** pain in sacrum, Helleb.

— spasmodic pain in sacrum, Mag-m.

**Confinement,** pain in sacral region after confinement, *Phos.*

**Cold** feeling and chills across loins and sacrum, Lil-tig.

— feeling in sacrum, *Dulc.*

**Coldness,** a sensation of coldness, numbness, and tension in sacrum, Carbo-v.

— a sensation of coldness in sacrum, Benz-ac.

**Couch,** pain in sacrum as if one had been lying on too hard a couch, *Merc.*

**Cough,** cutting pains in region of sacrum, hypogastrium, and groins with too early menstruation too profuse and pale, weak and with night cough, *Senecio.*

— shooting in sacrum when he coughs, Nit-ac.

— during cough lancinating pain above the left hip extending into the sacrum, Sul.

**Crawling** as from ants in sacrum, Sarsa.

— sensation with stitching pains in sacrum, *Graph.*

— beneath the skin of sacrum, Jatropha.

— aching in region of left sacro-iliac articulation with crawling in left sole as though it were asleep, *Coloc.*

**Cracking** in sacrum on walking, Zinc.

### SACRUM.

**Cracking** sound in sacrum extending to anus, Sul.
— in sacral joints on bending, Lyco.
**Creeping**, painless creeping in sacrum, Tarax.
**Crick** in back extends along spine to neck evening and goes to lower limbs, Agari.
**Cries** out if any attempt is made to touch sacrum, *Lob-i.*
**Crouching**, severe stitch in sacrum when arising from crouching down, Phos-ac.
**Cutting**, thrusts in sacrum, worse bending forward with tensive pain, Sambucus.
— before menses straining cutting and pain in sacrum as if contracted and bruised, particularly while sitting, less when walking, Mag-c.
— tearing low down at both sides of sacrum, Mez.
— a uterine hemorrhage that comes with every stool, with cutting in abdomen and pains in sacrum and loins, Iodium.
— pain in sacrum in morning after rising, and in evening before going to sleep, only when moving and bending down, not when standing upright, Petrol.
— severe pain in sacrum like a cutting through, during movement and at rest, so he could neither stand, walk, nor lie down, Hepar.
— in the epigastrium as after a laxative, extending into sacrum in the morning, Mag-m.
— pains in sacrum in mornings, All-sat.
— and pinching in stomach toward the sacrum and the left side, Nat-c.
— burning and scraping in the sacrum, Nat-c.
— in sacral region with pain, Mag-m.
— severe in sacrum at the least motion, extending into the calves and feet, so that he cannot walk nor stand or lie down, Zinc.
— pain in outer left side of sacrum, shooting up and down, *Kali-bichrom.*

### SACRUM.

**Cutting** pain across sacrum and left hip, also in muscles just above sacro-iliac symphysis of left side, Gambog.

— pressure in sacral region, Mag-m.

**Day,** pain in sacrum all day, *Lil-tig.*

— during menses violent pain in sacrum only during day, Nat-c.

**Darting,** sensation when sitting, as though something were darting through back toward sacral vertebræ, Aspar.

**Digging,** shooting pain in left side near sacrum, Dulc.

— as if bruised just above the sacrum when sitting after a long walk; better by continued walking and returning when standing still or sitting, Ruta.

**Dinner,** weak in sacrum, as if paralyzed in loins; could neither stand right nor walk; he feels best when lying down all the day; it is worse after dinner, Nat-m.

**Drawing** pressure in sacrum, extending to muscles on inside of ilium when standing, Sambucus.

— from sacrum to neck, causing trunk to bend forward, *Ars.*

— fine tearing, drawing from middle of sacrum toward lumbar vertebræ, Mur-ac.

— pain in sacrum when standing; better by pressure, Ledum.

— in sacrum while sitting, Calc-c.

— painful drawing in sacrum, as if a heavy body was moving downward there, Baryta-c.

— pain in left sacral region in evening first day of menstruation, Zing.

— pain in sacrum, Stram., Sul., Diosc., Kali-c.

— pain in sacrum with weakness, Sul.

— pain in sacrum, and a sensation as if broken in walking, standing, and lying, Carbo-an.

— pain just before menses from hypogastrium into sacrum, Carbo-veg.

— pain in sacrum for seventeen days, Lyco.

— pressive pain in sacrum down to coccyx, Carbo-veg.

— pain in sacrum toward evening, Nit-ac.

— pain in back and sacrum, *Chelid.*

SACRUM.

**Drawing** from sacrum to right side of scrobiculum, *Chelid.*
— stitches in sacrum when standing, with drawing through lumbar region, Con.
— in sacrum, *Thuja*, *Ant-c.*
— violently drawing pressive pain on left side near sacrum, Mez.
— a pain in sacrum drawing hither and thither, worse by walking, Hepar.
— severe writhing pains in sacrum and bowels, radiating upward and downward until whole body and even fingers and toes become enveloped in spasms, so severe as to elicit shrieks, *Diosc.*
— severe constant drawing in sacrum, alternating with throbbing, relieved by lying down, Kali-c.
— pain in sacrum on moving, like soreness or like a spasmodic drawing, Sul-ac.
**Dragging**, prolapsus uteri with dragging, aching pain in sacrum, *Helon.*
— backache, with dragging in sacrum and stringy colorless leucorrhœa, beginning with walking, Aletris.
— pressing pain over sacrum, and at same time over hips, burning pressure on sacrum extending up across dorsal region and under scapula, like that produced by sewing, *Sepia.*
— from sacrum toward rectum, Calc.
**Dull** pain in sacrum when stooping, Borax.
— pain in sacrum, *Sepia.*
— constant dull, heavy pain in sacrum and abdomen, extending to thighs and legs, *Sepia.*
— constant dull pain across sacrum and hips, *Æsc.*
— pain in sacrum and hips, Ham.
**Enuresis** with cutting scalding in the urethra and spasmodic pain in the sacrum, Phos-ac.
**Eruptive** nodules, sometimes itching and sometimes painful on occiput, the sacrum and nates, Lyco.
**Evening,** drawing pain in sacrum toward evening, Nit-ac.

### SACRUM.

**Evening,** pain as from weakness in sacral region after lying down in the evening, Zing.

— painful throbbing in the evening after going to bed in sacrum, Nat-m.

— bruised feeling in the sacral region in the evening, Sarsa.

— bruised pain in sacrum in every position in evening, during menses, Nitrum.

— paralytic sensation in sacrum in the evening, Mag-m.

**Excoriation,** pain in sacrum as from excoriation and a bruise, Mag-m.

— rheumatic sensation in neck extending to sacrum, worse when going to stool, as if skin was excoriated, Ver-a.

**Exercise,** menses two days too early, very thin and of short duration and with violent unusual pains in sacrum, which went off during exercise, Graph.

**Expiration,** shooting pain in sacral bone at every expiration, Sul.

**Eyes,** heat in the eyes, pain in sacrum and great anxiety, Nit-ac.

**Face,** pain in sacrum, so that he cannot lie on his back but must lie on his face, Nit-ac.

**Fall,** pain in os sacrum as from a fall, *Kali-iod.*

**Fatigue** and tickling pain above sacrum, Kali-c.

— pressing pain as from fatigue in sacrum in evening, *Puls.*

— pain in sacrum as from fatigue when stooping and when leaning back while sitting, Hepar.

**Feeling,** of paralysis in sacrum, *Kalmia.*

— bruised feeling in sacral region, Sabad.

**Feet,** pain in sacrum extending into feet, Lyco., Phyto.

**Fever,** violent pain in sacrum, with fever, *Lob-i.*

**Flatus,** pain as from inflation in sacrum in the morning in bed with a feeling as if great bubbles were accumulating in sacral region with tenesmus, all of which vanishes on the emission of flatus, Kali-c.

— hasty urging but only an ordinary stool coming twice after

SACRUM.

previous shooting and pinching in abdomen and going from there backward to the sacrum as from flatus in morning after awaking, Nitr.

**Flatus**, pain in sacrum as from flatus, Agnus-c.

— pressure in the abdomen on the sacrum as from flatus which passes off sparingly, affording some relief, Phos.

**Fluttering**, sense of fluttering-like movements of a watch commencing in region of sacrum and gradually arising to occiput, *Oleum-jec.*

**Forced**, pain in sacrum and bearing down as if parts would be forced out, worse when moving, Secale.

**Forcing**, pinching about the navel and forcing down toward the sacrum, then sudden urging to stool and a soft yellow stool with a piece of tape-worm, Mag-m.

**Formication**, intense formication in sacrum, Stann.

**Fracture**, pain as from fracture in sacrum, Nat-m.

**Fullness** in head and pressure in sacrum while sitting, with drowsiness at same time, Borax.

**Genitals**, sore pain in abdomen with pressure toward genitals as with the menses and pain in the sacrum, Kali-c.

**Gnawing** ameliorated by rubbing in small of back and sacrum, Phos.

— in sacrum in evening, Canth.

— pain in sacrum and the whole back in the evening after lying down, seemingly in the marrow, extending to the neck, so that she cannot sleep for pain and has to toss about continually, Mag-m.

**Griping** in sacrum worse by standing and better by walking, Merc.

— severe and twisting in sacrum as with tongs, and then also pain in arms and feet as if it would turn them outward, Graph.

**Guggling**, dull " guggling " in sacral bones, Angustura.

**Headache** alternately with a broken sensation in the sacrum and lumbar region, worse by sitting, better by walking or standing, Melilotus.

### SACRUM.

**Heaviness**, during menses, pain in sacrum! like heaviness, Kali-c.

— pain in sacrum, like a great heaviness, arising suddenly when sitting, and ceasing when moving about, Nat-c.

— in sacrum and loins as from a cold, Baryta-c.

**Heavy**, dull pain in region of sacro-iliac symphisis and a dull pain in liver region, Diosc.

**Heels**, acute squeezing pain in the left thigh in the morning on awaking, on turning over it extends to sacrum and terminates with shooting pains in the heels, Nitrum.

**Intermitting**, during menses frequent, but intermittent pains in sacrum, Mag-c.

**Intolerable** pains in sacrum, Calc-c.

**Iron**, pain in back as if hot iron was thrust through sacral vertebræ, Alum.

— sensation in sacrum as if a red-hot iron passed up spine to the atlas, around occiput, over eyes from right side, stopping at left ear, leaving a charred feeling; to perform the passage it takes six hours, Can-ind.

**Itching** on lower part of sacrum, Kali-c.

— and burning in sacrum and below right patella, Kali-c.

— eruptive nodules, sometimes itching and sometimes painful on the occiput, the sacrum and nates, Lyco.

— single itching stitches in sacrum, Caustic.

— severe itching on sacrum in bed, in evening, Nat-m.

— worse by scratching on outer side of thigh, on sacrum and hips succeeded by burning, Mag-m.

— and pricking in sacrum when walking, *Merc.*

— on back, chest, the dorsum of left foot, and on the sacrum, not going off by scratching, Mag-m.

— burning in sacrum above nates, Mag-c.

**Jerking**, shooting pain in sacrum, Euphorb.

— sticking, piercing, jerking in sacrum, and back arresting breath, Caustic.

— after confinement aching pain in sacrum, very suddenly going

SACRUM.

down limbs to knees like in the bone, passing down gradually to the ankle and returning again to sacrum; jerking pains in different parts of the body, Phyto.

**Jerking** pains extending from left sacrum to anus, Calc-c.

— in sacrum, Fluor-ac.

**Jerks,** painless jerks in sacrum in evening in bed, Sul.

— painful jerks in sacrum during many movements, Petrol.

— intermitting tearing in sacrum after rising from stooping; while standing still it quietly draws in jerks, Phos-ac.

**Labor,** during the menses pain in abdomen, straining and pressure like labor pains, pain in back and anxious pains in sacrum, commencing with tickling, Graph.

— like pains across sacrum, *Kali-c.*

— spasm in sacrum and rectum like labor pains, Lyco.

**Lame** feeling in region of sacrum, *Sil.*

**Lameness** in sacrum in morning when rising, Nat-m.

— and numbness in sacrum, *Calc-Phos.*

**Lancinating,** during cough lancinating pain above left hip extending into sacrum, Sul.

**Lancination** in sacrum, *Zinc.*

**Lassitude,** pain in sacrum extending to hips and thighs to below knees with weakness and lassitude when moving, when going upstairs, as if limbs would refuse to act before reaching top, Sepia.

— severe aching in sacral region from walking a short distance, followed by nausea and lassitude, Con.

— severe pain in sacrum after walking a little, Con.

— pain in sacrum when lying upon it with great lassitude, Lyco.

**Leucorrhœa,** backache, with dragging in sacral region and stringy, colorless leucorrhœa, beginning when walking, Aletris.

— sensation as from a bruise in the sacrum toward evening for several hours, with discharge of leucorrhœa, Caustic.

— pain as after a violent blow in the left hypochondrium; with

SACRUM.

pains in sacrum, often so intense that she could not lie down, followed by leucorrhœa of eight days' duration; this and the pain in the sacrum ceased only with the appearance of menses, Nitrum.

**Lie**, pain in sacrum so that he cannot lie on his back, but must lie on face, Nit-ac.

— severe pain in sacrum like a cutting through during movement and at rest so that he could neither stand, walk nor lie down, Hepar.

— severe pain in sacrum so she could neither sit nor lie down, Calc-c.

**Lifting,** pain in sacrum from heavy lifting, Calc-c.

— pain in sacrum when lifting, *Sang-c.*

— like a gathering in a small spot in sacrum, a steady throbbing pain most felt at night and hinders sleep, violent aching pain, better in day when up and walking about, but unable to do any lifting, *Kali-b.*

— violent pains in sacrum when lifting a slight load, takes her breath, Anag-ars.

— pain in sacral region, back and neck after overlifting or feeling as if wrenched, *Calc-c.*

**Lying**, lameness in sacrum in morning when rising, weak in sacrum, as if paralyzed in loins; could neither stand right nor walk; he feels best when lying down all the day; it is worse after dinner, Nat-m.

— severe constant drawing in sacrum, alternating with throbbing, better by lying down, Kali-c.

— pressive, tensive sensation in sacrum, deep internal when severe, with feeling in bone as pressed asunder, worse when sitting and when lying, *Berb.*

— pain in sacrum as if one had been lying on a hard couch, *Merc.*

— pain in sacrum as if bruised, when starting to move after lying down, Dig.

— pain in sacrum when lying on back in morning, Ign.

SACRUM.

**Lying**, gnawing pain in sacrum and the whole back in evening after lying down, seemingly in the marrow, extending to the neck, so she cannot sleep for the pain, and has to toss about continually, Mag-m.

— drawing pain in sacrum and a sensation as if it were broken, in walking, standing and lying, Carbo-an.

**Menses**, a drawing pain from hypogastrium to sacrum just before menses, Carbo-veg.

— bruised pain in sacrum in every position in evening during menses, Nitrum.

— delayed five days with pains in abdomen and sacrum, Sul-ac.

— sore in sacrum before menses, *Spong.*

— fourteen days too early with pain especially violent in sacrum, worse when sitting, better while walking, Mag-c.

— violent pain in sacrum during menses, but only by day, Nat-c.

— four days late and of shorter duration than usual, with violent pain in sacrum, Mag-m.

— during normal menses, pain in sacrum, Iodum.

— during menses, pain in sacrum like heaviness, Kali-c.

— during menses, severe pains in sacrum mornings on rising from bed so she could not move, Lyco.

— two days too early, very thin, of short duration, with violent, unusual pains in sacrum, which went off during exercise, Graph.

— at the appearance of menses, a violent pain in the sacrum for an hour, Nit-ac.

**Menstruation**, chilliness and pain in sacral region during menstruation, *Puls.*

— drawing pain in left sacral region in evening first day of menstruation, Zing.

**Morning**, lameness in sacrum in morning, with weakness when rising as if paralyzed in loins; could neither stand right or walk; feels best when lying down all day; is worse after dinner, Nat-m.

— bruised pain in sacrum from morning till afternoon, Mag-c.

### SACRUM.

**Morning,** pain in sacrum in morning as if she had received a blow, Nitrum.

— cutting pains in sacrum in morning, All-sat.

— sensation in morning as if sacrum were being pressed inward from both sides, Kali-c.

— pain in sacrum in morning; on awaking she could not remain in bed; she must get up, Nitrum.

— pain in sacrum as beaten; in morning worse by stooping, not better by rising again; extending from time to time in renal region; better by walking in open air, worse on right side, Tellur.

— pain in lumbar and sacral region in morning on waking, Erigeron.

— pain in sacrum morning on rising; better in evening, Lil-tig.

— drawing pain in sacrum; better by moving in morning, Lil-tig.

— pain in sacrum at once in morning after rising, Calc-c.

— sacral region and back; feel bruised in morning on rising, Callad.

— colic at night for two hours, with subsequent stool; then in morning pain in sacrum and chest, and afternoon all the limbs feel bruised, Kali-c.

— lying on back; pain in sacrum in morning, Ign.

**Motion,** she painfully feels every motion of the body in the sacrum, Caust.

— stitches in sacrum; worse when sitting than when in motion, Bar-c.

— shooting pain in sacrum, so that he must keep perfectly quiet; worse by every motion, Coloc.

**Moving,** restless, sleepless night on account of violent pains in sacrum, which force him to be constantly moving Mag-m.

— severe pain in sacrum almost only when moving, so that he can hardly walk; seems to be in the bone, Nit-ac.

— pain in sacrum, passing on both sides around the pelvis forward toward the genitals; worse at night and when moving, Sarsa.

SACRUM.

**Moving** pain in sacrum on moving like soreness or like a spasmodic drawing, Sul-ac.

— pain in sacrum extending through the hips and thighs to below the knee, with weakness and lassitude when moving, especially when going upstairs, as if limbs would refuse to act before reaching top, Sepia.

— sprained pain in hip beside sacrum when moving, Petrol.

**Nausea,** severe pain in sacrum after walking a little, then nausea and lassitude, Con.

**Needle** like pains in sacrum and in hip bones, Chimaphilla.

**Night,** pain in sacrum at night, and on the top of that shoulder on which he is lying, Sil.

— tearing and burning in sacrum and hips afternoon and night, Mag-m.

— pain in sacrum and back at night so violent that she could not lie still, Mag-c.

— violent pains in sacrum at night, they awakened her and did not allow her to sleep again, Nitrum.

— a feeling of prostration in sacrum at night, *Lith-c.*

— pain in upper part of sacrum and pelvis at night is worse, *Helon.*

— pain in sacrum passing on both sides around the pelvis forward toward the genitals worse at night and when moving, Sarsa.

— bruised pain in sacrum at night, at 3 A. M. she could not turn over, Nitrum.

**Nose,** pain as from a bruise in sacrum when blowing nose, Dig.

**Numbness** and lameness in sacrum, *Calc-phos.*

— a sensation of coldness, numbness, and tension in sacrum, Carbo-v.

**Numb** pain from sacrum down legs, *Graph.*

**Pain** in sacrum as after long stooping, Dulc.

— violent bruised pain in sacrum from afternoon till evening, Mag-c.

— in sacrum, Chelid., *Sepia.*, *Sul-ac.*, Nit-ac., Mez., Borax., *Calc-c.*, Ver-vir., *Podo.*, Lyco., Mag-m., Carbo-an., *Magnolia*, *Abrot.*, Absinth.

SACRUM.

**Pain** in lower part of sacrum, Carbo-an.
— begins in sacrum and goes into left hip, tensive as if muscles were too short, Caust.
— in sacrum when straightening himself after long stooping, Nat-m.
— sore pain in sacrum even while at rest, also when not touched, Nat-c.
— as if flesh about lower part of sacrum was detached, Lyco.
— especially in hip and sacrum with prolapsus uteri, Æscul.
— in sacrum, cannot lie on either side, Nat-s.
— bruised pain in sacrum and small of back, *Nat-s*.

